# Formation and shape-control of hierarchical cobalt nanostructures using quaternary ammonium salts in aqueous media

**DOI:** 10.3762/bjnano.8.53

**Published:** 2017-02-23

**Authors:** Ruchi Deshmukh, Anurag Mehra, Rochish Thaokar

**Affiliations:** 1 Department of Chemical Engineering, Indian Institute of Technology Bombay, Powai, Mumbai 400076, India

**Keywords:** Brownian motion, cobalt nanoplates, electron microscopy, hierarchical nanostructures, magnetic moment, tetramethylazanium hydroxide (TMAH)

## Abstract

Aggregation and self-assembly are influenced by molecular interactions. With precise control of molecular interactions, in this study, a wide range of nanostructures ranging from zero-dimensional nanospheres to hierarchical nanoplates and spindles have been successfully synthesized at ambient temperature in aqueous solution. The nanostructures reported here are formed by aggregation of spherical seed particles (monomers) in presence of quaternary ammonium salts. Hydroxide ions and a magnetic moment of the monomers are essential to induce shape anisotropy in the nanostructures. The cobalt nanoplates are studied in detail, and a growth mechanism based on collision, aggregation, and crystal consolidation is proposed based on a electron microscopy studies. The growth mechanism is generalized for rods, spindles, and nearly spherical nanostructures, obtained by varying the cation group in the quaternary ammonium hydroxides. Electron diffraction shows different predominant lattice planes on the edge and on the surface of a nanoplate. The study explains, hereto unaddressed, the temporal evolution of complex magnetic nanostructures. These ferromagnetic nanostructures represent an interesting combination of shape anisotropy and magnetic characteristics.

## Introduction

The synthesis of ferromagnetic nanomaterials with complex functional architectures has seen rapid development during the past decade [1–3]. Cobalt nanostructures form an important class of ferromagnetic materials because of distinctive magnetic properties such as an exponential dependence of the magnetization relaxation time on the volume of a particle [4], high susceptibility, many available crystal structures [5] and the uniaxial magnetic axis in hexagonal close-package (hcp) cobalt [6], which is not observed in other ferromagnetic materials such as nickel and iron.

Synthesizing anisotropic and hierarchical nanostructures of cobalt is an effective strategy for tuning its electronic, magnetic and crystal properties. The interest in these nanostructures lies in comprehending the physical and chemical forces that drive the hierarchy. These nanostructures also show novel properties that are different from their bulk or discrete counterparts and could be of immense use for catalysis [2], magnetic recording media [7], magnetic sensors [8], and biomedical science [9]. For example, consider the case of the morphology-dependent catalysis by cobalt nanostructures. It is understood that the synthesis of nanoparticles with well-controlled shape and a well-defined reactive crystal plane is critical for efficient catalytic performance. For example, appropriate loading and dispersion of cobalt nanoparticles on a support yields increased active sites in a Fischer–Tropsch synthesis [10]. Another example is the decomposition of ammonium perchlorate [11] in which the addition of cauliflower and snowflake cobalt particles (BET surface areas of 7.23 and 1.65 m^2^/g, respectively) reduces the decomposition temperature by 120 °C and 150 °C respectively, thereby increasing the decomposition heat. The greater catalytic effect of snowflake particles, despite the smaller BET surface area, is attributed to the surface defects developed during their liquid-phase reduction. Recent studies also emphasize that the lattice arrangement in an anisotropic dendritic cobalt nanocrystal, via adsorption and chemical catalytic oxidation, increases the efficacy for sewage water treatment [12–13] compared to its spherical counterparts.

In addition to the catalytic activity of cobalt, its magnetic characteristics are also important. Cobalt nanostructures with defined geometry and high coercivity are most desirable for magnetic storage applications [7]. Therefore, it is important to develop synthesis methods that yield cobalt nanostructures with well-controlled shape and size. This formulates the primary objective of the present work. In this study, we report the formation of various nanostructures of cobalt such as nanoplates, nanorods, nanospheres and nanospindles with well-defined crystal planes by performing an aqueous syntheses at ambient temperature.

Various synthesis strategies have been used in the literature to obtain cobalt nanostructures of desired size and shape such as spherical nanoparticles [14–15] synthesized by high-temperature chemical reduction while controlling the pH value, cobalt–polymer composite tubes [16] formed by using alumina templates, cobalt cubes [17] produced in imidazolium ionic liquid or by thermal decomposition [18]. An alternative way to obtain complex hierarchical nanostructures is the controlled assembly of primary building blocks using electric and magnetic forces, capillary effects, and surface defects. Fascinating shapes such as, rice grain-like structures were formed by strong magnetic dipolar interactions during the evaporation of 6 nm cobalt nanoparticles on oriented pyrolytic graphite [19]. Cobalt wires were obtained by the reduction of cobalt salt at high temperatures [20], and discs were produced by applying high temperature in a mixed surfactant system of oleic acid and trioctyl phosphine [21]. The formation of nanoplates has been observed in alkaline environments [6,22–23], and nanoflowers were obtained in the presence of small ionic species such as the hydroxide ion (OH^−^) in an autoclave environment [24]; or by using a chelating agent [25]. A similar mechanism, based on hydroxide ion-assisted nanocone formation, has been reported [26], wherein the growth of the nanocones is initiated by a screw dislocation present on the copper substrate. Also, chains composed of microplates [27] have been formed by using mixed solvents such as ethylene glycol and ethylenediamine in a solvothermal approach.

The synthesis strategies mentioned above are specific to a certain dimensionality of the nanostructures, with a limited possibility for its variation from zero-dimensional spheres to hierarchical nanostructures. In this context, the present study contributes in several new ways. (i) We propose a new method to form hierarchical nanostructures of cobalt by an aqueous route under ambient conditions, using quaternary ammonium salts; (ii) a variety of shapes, from zero-dimensional spheres to hierarchical interlocked nanoplates and spindles, is obtained through small variations of the protocol; (iii) nanostructures are obtained only by the precise control of the synthesis parameters without the addition of surfactants or applying external magnetic fields; (iv) we use electron microscopy to capture important modes of aggregation and growth for highly ordered hierarchical nanostructures; (v) we identify the importance of crystal twinning and various forces necessary for the formation of anisotropies. This is made possible by a detailed study of a cobalt–tetramethylammonium hydroxide (TMAH) system that forms nanoplates under ambient conditions. The understanding of this system is extended to obtain other anisotropic shapes formed by using various quaternary ammonium compounds. The quaternary ammonium salts used here are also commonly used, in their pure form as ionic liquids, as solvents [28]. In this form they exhibit structures of high directionality and were recently used as shape-selection agents [17].

## Experimental

### Materials

Analytical grade reagents were procured and used without further purification. Cobalt chloride hexahydrate (CoCl_2_·6H_2_O), sodium borohydride (NaBH_4_), and ammonium hydroxide (NH_4_OH) were procured from Merck Chemicals, India. The quaternary ammonium salts, tetramethylammonium hydroxide (TMAH, 25% (w/w), aqueous), tetraethylammonium hydroxide (TEAH, 35% (w/w), aqueous), and tetrabutylammonium hydroxide (TBAH, 25% (w/w), aqueous) were procured from Sigma-Aldrich Chemicals GmbH. In all the experiments, MilliQ water with a conductivity of σ = 0.0016 S/m was used and the temperature was maintained at 27 °C unless mentioned otherwise.

### Synthesis

In a typical procedure, cobalt chloride hexahydrate (CoCl_2_·6H_2_O) was reduced by sodium borohydride (NaBH_4_) to obtain spherical cobalt seed nanoparticles with an average size of 29 ± 3 nm. These seeds were washed with an ethanol–water mixture, followed by magnetic decantation and ultrasonication in the presence of either TMAH, TEAH, TBAH or NH_4_OH. A detailed description can be found in Supporting Information File 1. Figure S1 (Supporting Information File 1) shows the presence of cobalt in the energy dispersive X-ray spectrum. The high-resolution micrograph and the selected-area electron diffraction (SAED) pattern in Figure S2 (Supporting Information File 1) indicates the crystalline nature of the seed particles. The lattice observed here shows a spacing of 0.12 nm corresponding to hcp Co(110). Furthermore, the size distribution of a typical sample of cobalt seed particles is shown in Figure S3 (Supporting Information File 1).

### Characterization

The intermediate stages of the growth and aspects such as shape, size and growth direction were investigated by using field emission gun transmission electron microscopy (FEGTEM), high-resolution transmission electron microscopy (HRTEM), and field emission gun scanning electron microscopy (FEGSEM). FEGTEM investigations were carried out by using a JEOL, JEM2100F operated at 200 kV, and the HRTEM was carried out using a JEM 2100 with a LaB_6_ filament operated at 200 kV. A JEOL JSM-7600F FEGSEM, operated at 5, 10 and 15 kV, was used to obtain information about morphology and topology of the nanostructures. Microscopy samples were prepared by placing a small drop of the sample on a carbon-coated copper 200 mesh grid and subsequent vacuum drying at room temperature for 12 h. Energy dispersive X-ray spectroscopy (EDS) spectra were obtained during FEGTEM imaging to confirm the presence of cobalt and to detect traces of other elements. X-ray diffraction (XRD) measurements for nanostructures collected and dried in a vacuum desiccator were carried out on a Rigaku D-max 2000/JADE 6.0 X-ray diffractometer using Cu Kα_1_ radiation. The magnetic properties of the samples dried in a vacuum desiccator were determined by vibrating sample magnetometry (Quantum Design, USA). The samples were also analyzed using X-ray photoelectron spectroscopy (XPS, VG Micro Tech ESCA-3000) using non-monochromatic, Al Kα radiation (*h*ν = 1486.6 eV) with a spectral resolution of 0.1 eV.

## Results and Discussion

### The role of TMAH in the formation of nanoplates and nanorods

During the formation of nanoplates four important observations were made: (i) Nanoplates are formed only at a certain concentration of hydroxide ions; (ii) after replacing the hydroxide anion in tetraalkylammonium hydroxide with other ions (e.g., chloride ions) nanoplates do not form; (iii) nanoplates are formed only under ambient growth conditions, at higher temperatures rods are obtained; (iv) steric hindrance by the alkyl group of the ammonium salt changes the shape of the nanostructures.

**Figure 1:**
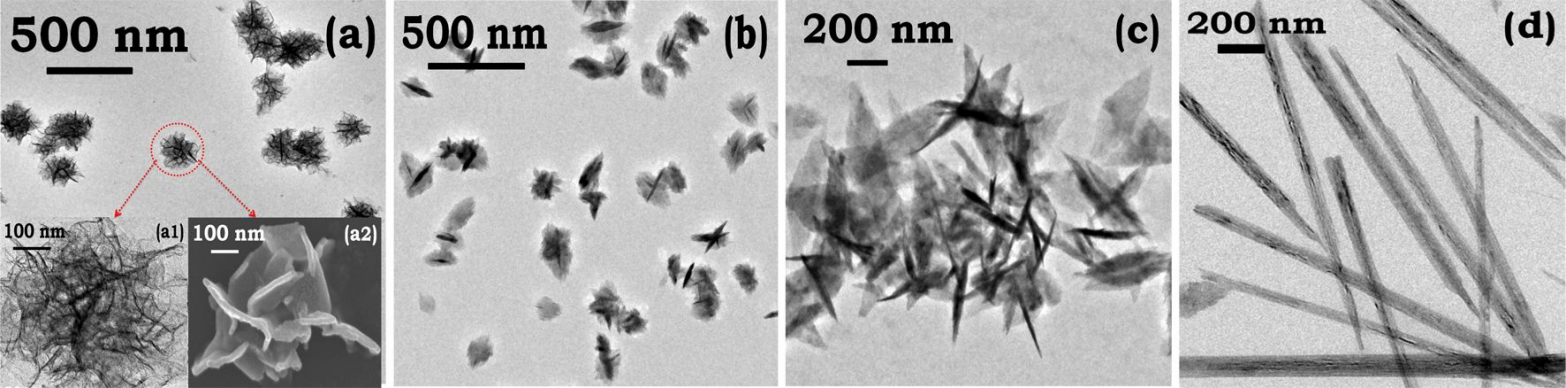
The concentration of TMAH and the growth temperature play a critical role in controlling the morphology of cobalt nanoplates. FEGTEM micrographs captured after 45 min: (a) Sample A (*c*_TMAH_ = 0.01 M) size of single flower = 250 nm, insets (a1) and (a2) show flower-like structures made of cross-linked plates; (b) Sample B (*c*_TMAH_ = 1 M) nanoplate diameter = 200 nm, thickness = 25 nm; (c) Sample C (*c*_TMAH_ = 2.7 M) nanoplates with diameter = 250 nm, thickness = 30 nm; (d) nanorods obtained at 50 °C with length = 1400 nm and diameter = 110 nm.

In order to obtain nanoplates with controllable crystallographic facets, the growth kinetics should be carefully controlled by choosing an appropriate TMAH concentration in the growth solution. The concentration of added TMAH determines the morphology of the nanostructures. The concentration dependency here is related to the availability of hydroxide ions. The TEM micrographs in (Figure 1), show that too low a concentration of TMAH (0.01 M, pH 7.5) results in flower-like nanostructures (Figure 1a). These flowers are faceted nanoplates interconnected to each other through a common center (Figure 1a, insets a1 and a2). An increase in the concentration of TMAH (0.1 M, pH 10) yields nanoplates of small size (Figure 1b). Further increase in the concentration (2.7 M, pH 12) shows increased stability, well-defined geometry and interlocking (Figure 1c), and is further examined in detail. This observation is in agreement with previous studies that suggest that shape anisotropy is observed only above a certain concentration of hydroxide ions [23–24,29]. EDS measurements of well-formed nanoplates showing the presence of cobalt is are given in Figure S4 (Supporting Information File 1).

Under similar experimental conditions, the addition of tetramethylammonium chloride (TMACl) to the seed solution did not yield nanoplates (Figure S5, Supporting Information File 1), indicating that presence of hydroxide ions is critical to obtain nanoplates. Interestingly, under identical experimental conditions, with the exception of an elevated temperature (50 °C) nanorods are obtained (Figure 1d). We therefore hypothesize that a synergistic effect of OH^−^ adsorption [23–24,29–30], inherent magnetic moment, controlled aggregation and growth temperature are the factors influencing the geometry of the cobalt nanostructures. The experimental parameters for the cobalt–TMAH system are summarized in (Table 1).

**Table 1:** Effect of various parameters on cobalt-nanoplates formed in the presence of TMAH: All values are reported after 45 min; the concentration of cobalt precursor and reducing agent were 0.4 M and 0.01 M, respectively. Viscosity of TMAH = 2.8 cSt at 25 °C, ionic radii: TMA^+^ = 2.9 Å, and OH^−^ = 0.95 Å.

growth temperature	*c*_TMAH_	shape	typical size	
(°C)	(M)		*d* (nm)	*t* (nm)

27 ± 2 (room temperature)	0.01	flower-like structures	—	—
	1	small nano-plates	200 ± 3	25 ± 5
	2.7	interlocked nano-plates	250 ± 4	30 ± 5
50	2.7	rods	1400 ± 8	110 ± 3

**Figure 2:**
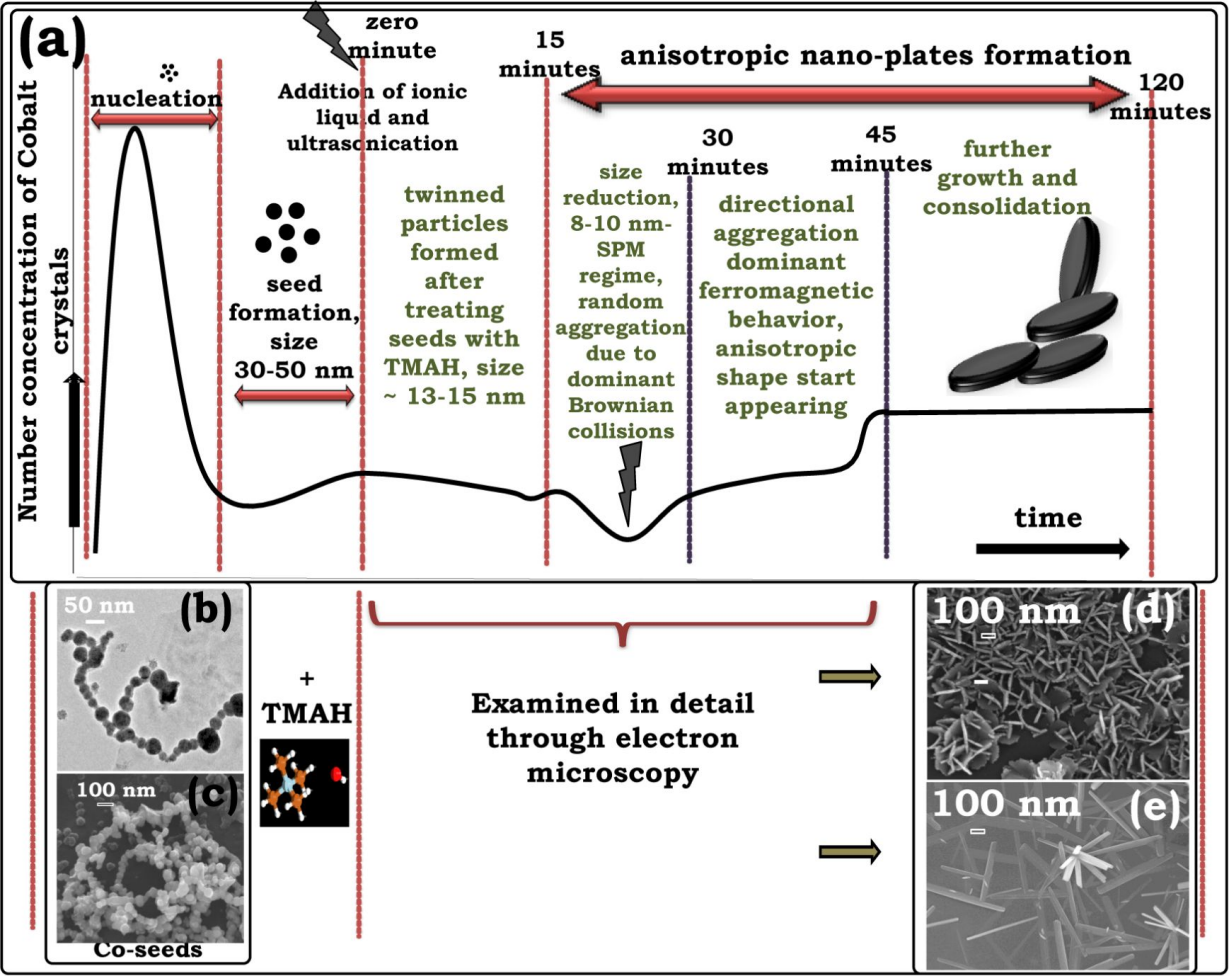
Schematic representation of growth and interlocking of cobalt nanoplates. (a) The growth curve shows that the formation of nanoplates can be divided into nucleation, kinetically driven seed formation, and growth; (b,c) FEGTEM and FEGSEM images of spherical cobalt seeds; (d) hierarchical long-range ordered crystalline nanoplates observed after 120 min, (e) nanorods obtained after 45 min.

The formation of nanoplates and nanorods (Figure 1c,d) was observed by capturing electron micrographs at definite times during the growth process. Analyzing various micrographs obtained, we define important steps for the formation of these anisotropic structures in Figure 2. The process has three important stages: (i) nucleation, (ii) seed formation, and (iii) slow crystal growth (Figure 2a). The seed particles initially have size of around 30-50 nm (Figure 2b,c), and when treated with TMAH, undergo various size and structural transformations given in the growth curve in Figure 2a. We emphasize here that the growth stage appears to be of prime importance since it yields distinctly shaped anisotropic structures through driving forces of different magnitude, in this case temperature. A detailed analysis of the growth regime is presented in the following to clearly explain various aspects such as twinning, size reduction of particles, lattice parameters, and time scales of growth encountered in the growth process.

### Growth and morphology

The growth of nanoplates obtained under ambient conditions and under zero shear is discussed first. The critical steps for the formation of nanoplates are the twinning of seed crystals, the size reduction of particles and their controlled assembly to form two-dimensional nanoplates.

**Figure 3:**
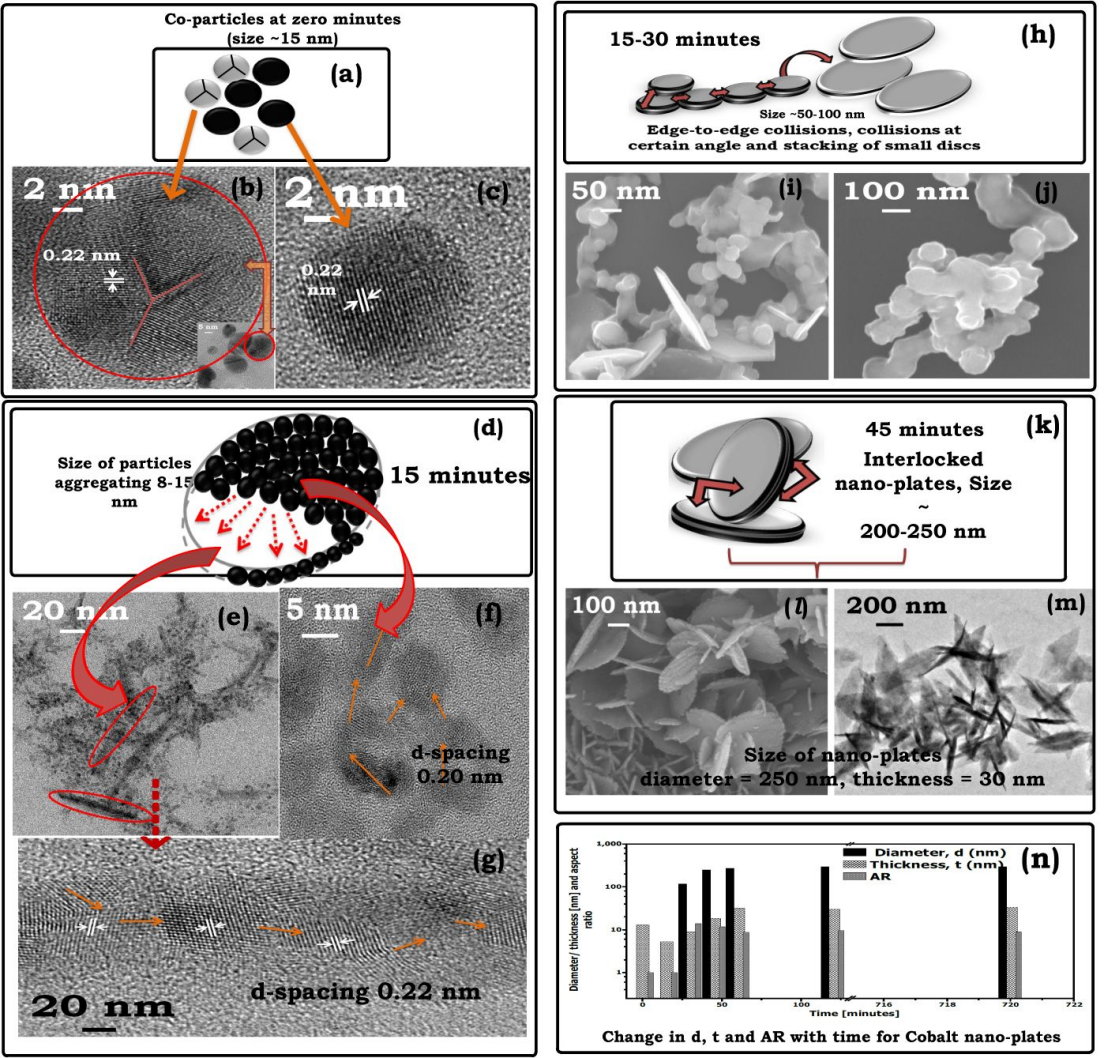
Schematic showing the growth of nanoplates. (a) Simultaneous presence of twinned and single- crystal nanoparticles at “zero time”; (b) high-resolution image of twinned nanoparticles; (c) high-resolution image of single-crystal nanoparticles, both observed at “zero time”; (d) the illustration shows how different modes of aggregation of tiny particles form the upper and side planes of the nanoplates after 15 min; the size of the nanoparticles drops from 15–20 nm to 8 nm; (e,f) low- and high-resolution micrograph, showing the spread of small nanoparticles with linear aggregation; (g) Brownian-motion controlled random aggregation of nanoparticles; (h) edge-to-edge collision of small nano-discs in the transition regime; (i) FEGSEM micrograph showing edge-to-edge aggregation after 15–30 min; (j) FEGSEM micrograph showing small nano-discs in the vicinity of bigger nanoplates after 15–30 min; the time interval of 15 min marks the initiation of shape anisotropy; (k) interlocked nano-plates appear first after 45 min; (l,m) FEGSEM and FEGTEM, respectively, micrographs showing interlocked nanoplates after 45 min; (n) length, diameter and aspect ratio distribution of nanoparticles as a function of time from 0 to 120 min.

The cobalt seeds are small spherical nanoparticles of ca. 30–50 nm that show no surface defects or faults (Figure S2, Supporting Information File 1). A mixed population of twinned and un-twinned spherical nanoparticles with an average particle of ca. 15 nm is obtained (Figure 3a) when these seeds are ultrasonicated followed by the instantaneous addition of TMAH. This is marked as “zero time” in the growth curve. The difference in lattice orientation across the particle–particle boundary (Figure 3b) results in a three-fold twinned structure [31] having a lattice spacing of 0.22 nm. A un-twinned crystal structure is shown in (Figure 3c). Additional micrographs supporting the twinning at zero minutes are provided in Figure S6 (Supporting Information File 1). Cobalt has a low stacking-fault energy [32] and an enhanced tendency to twin [33]. The coexistence of single and twinned nanocrystals indicates that various size and atomic rearrangements take place within the growth solution and, more importantly, form the basis for the generation of anisotropic particles [34].

Micrographs captured after further ageing for 15 min show that the particle size is reduced to 8–10 nm. The seeds ultrasonicated with TMAH undergo size reduction from 30 nm to 15 nm, and further to 8–10 nm over 15 min. The size reduction is attributed to etching by TMAH [35], which exposes favorable crystal planes. The (002) plane is possibly exposed because of this etching. Cobalt nanoparticles in the size range of 8–10 nm are superparamagnetic. Magnetic interactions are therefore absent and hence the regime is dominated by stochastic aggregation due to Brownian motion. The stochastic aggregation results in the formation of nanoclusters with larger size than at “zero time” possessing an increased magnetic moment. Figure 3d gives an impression of how the edge (thickness) and the lateral surface (diameter) of nanoplates is seen under the electron microscope. The corresponding electron micrograph (Figure 3e), shows an array of small nanoparticles forming an edge of a nanoplate. A high-resolution image for this aggregate illustrated in Figure 3f shows a lattice spacing of 0.22 nm, which is similar to the lattice spacing of twinned planes. A micrograph of an edge-to-edge aggregation (lattice spacing of 0.20 nm) that forms the lateral surface of a nanoplate is given in Figure 3g. The un-twinned planes form this lateral surface and because of their higher energy are more susceptible to the adsorption of TMAH, resulting in a disc-like aggregation. The assembly of these intermediate nanoclusters happens through continuous rotation and interactions of the primary particles. The translation and rotation occur until the primary particles find a suitable atomic site with compatible lattice arrangement that simultaneously satisfies the thermodynamic energy criteria of minimum free energy configuration [36].

In the transition regime, from 15 to 30 min (Figure 3h), we observe that the small nanoparticles almost disappear and intermediate clusters form by the assembly of nanoparticles. It is speculated that the growth and consolidation of these intermediate clusters happens due to minimization of magnetic anisotropic energy to achieve a stable crystal configuration. The nanoclusters observed in FEGSEM micrographs (Figure 3i) show the coexistence of small discs and larger nanoplates. Similarly, Figure 3j shows the stacking of small nanoplates in this transition regime. Well-formed nanoplates with interlocking (Figure 3k) first appear in micrographs captured after 45 min exhibiting hierarchical nanostructures (Figure 3l,m). The formation of these circular nanoplates can be explained by the tendency of two-dimensional objects to reduce their edge energy. The nanoplates typically have a diameter of 250 nm and a thickness of 30 nm after 45 min. The length, diameter, and aspect ratio (AR) distribution of the nanoplates is illustrated in Figure 3n.

It is important to mention here that FEGTEM and HRTEM, in normal modes of operation, allow access only to lateral size and shape of nanostructures limiting the information about the third-dimension which may thereby create a false impression about the shape of the nanostructures. For example, in Figure 3m, the nanoplates could be interpreted as nanoneedles because of the significant contrast variation. In fact, we had reported our preliminary results in [37], where we were led into interpreting the observed structures as nanoneedles. However, additional SEM micrographs showed that our presumed interpretation was erroneous (Figure 3l). The impression of needles is evoked by mass-thickness contrast, such that, the nanoplates standing upright on their edge appear brighter than the nanoplates lying perpendicular to the electron beam. Therefore, it is emphasized that a careful interpretation of electron micrographs is essential to avoid misleading conclusions and often SEM micrographs may be required for more accurate interpretation.

**Figure 4:**
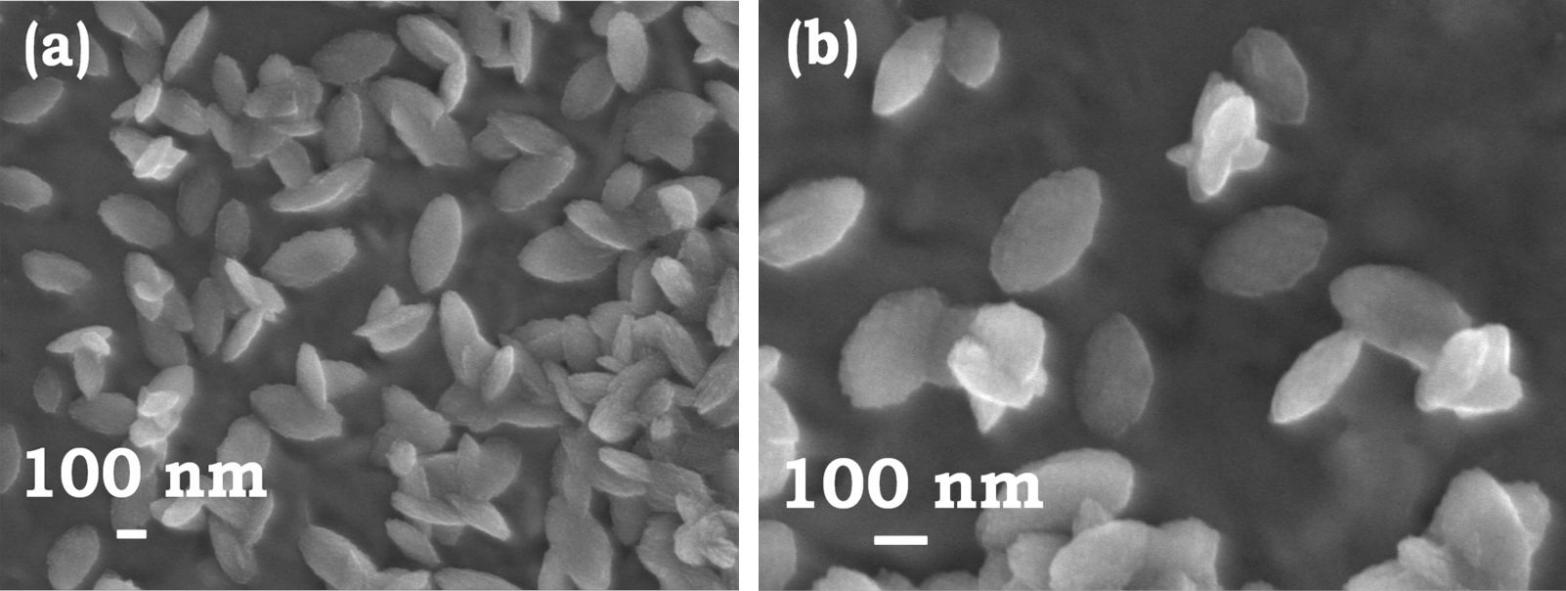
Cobalt nanoplates formed under ultrasonication. Micrographs captured after 45 min emphasize the importance of ultrasonic treatment to reduce the aggregation and interlocking tendency in nanoplates. Typical sizes: diameter *d* = 260 nm; thickness *t* = 34 nm.

As discussed above, the nanoplates here were formed by aging the Co-TMAH sample under zero shear. A controlled experiment was performed by allowing the growth of seeds treated with TMAH in an ultrasonication bath held at a temperature of 30 °C. The ultrasonication reduces the interlocking of the nanoplates (Figure 4). The typical size of the nanoplates is 260 ± 5 diameter and 34 ± 5 nm thickness. The reduced interlocking can be explained by the efficient dispersion of the nanoplates under sonication. The observation also reinforces the fact that the nanoplates are two-dimensional laterally confined structures and additionally, also provides a methodology to obtain separated nanoplates.

**Figure 5:**
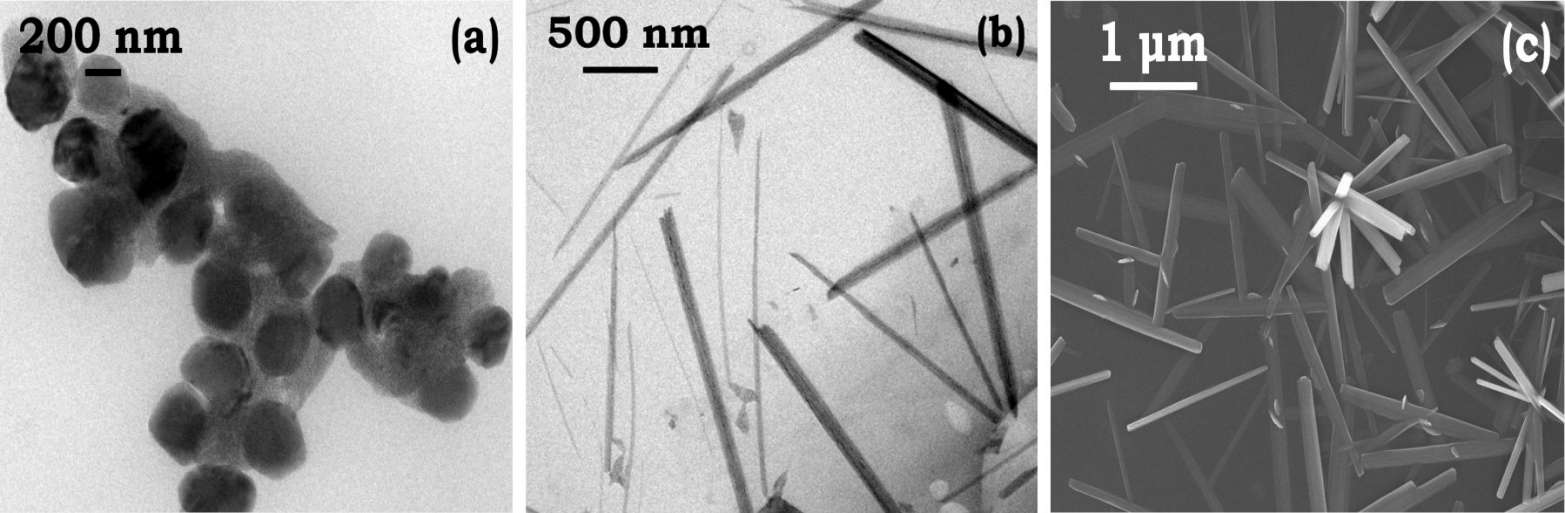
FEGTEM micrographs of cobalt-TMAH rods synthesized at 50 °C. (a) FEGTEM micrograph captured after 15 min, size = 105 nm; (b) FEGTEM micrograph captured after 45 mins, length 0 1400 ± 8 nm, diameter = 110 ± 3 nm. The cobalt rods formed by directional aggregation of nanoparticles obtained after 15 min. (c) FEGSEM micrograph of cobalt rods shows that rods are connected at 90°, however, a few nanorods are isolated as well.

It was found that the ambient growth temperature is critical for the formation of nanoplates. At higher temperatures (50 °C) under reflux conditions, a high yield of cobalt nanorods rather than nanoplates is obtained. Micrographs captured after 15 and 45 min show spherical nanoparticles of 105 nm diameter (Figure 5a), and rods of 1400 ± 8 nm length and 110 ± 3 nm diameter (Figure 5b). The presence of large aggregating spherical nanoparticles 150 nm, compared to 8 nm particles under ambient conditions, at initial time suggests that the effectiveness of TMAH to cleave and twin the seeds is lost. The aggregation of untwinned uncoated superparamagnetic nanoparticles results in large spherical nanoclusters, which then aggregate by magnetic interaction (ferromagnetic) by oriented attachment resulting in nanorods. The FEGSEM images (Figure 5c) show a persistent tendency of nanorods to interlock while a few isolated nanorods can also be seen.

In the following we explain the crystal structure and the effect of different parameters on the morphology of the nanoplates.

### Crystal structure

**Figure 6:**
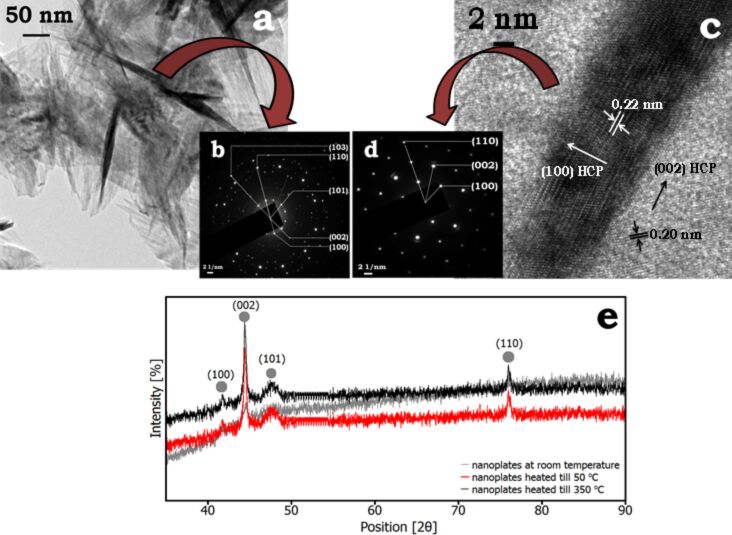
Crystal structure analysis of hierarchical nanoplates. (a,b) A group of nanoplates and the corresponding SAED pattern obtained by an electron beam of 30 nm diameter shows various planes of hcp cobalt; (c,d) high resolution image of a single nanoplate, the edge is normal to the direction of the electron beam, and the obtained SAED pattern at a spot focused with electron beam with 5 nm diameter; (e) XRD spectrum of fully grown cobalt nanoplates (after 120 min).

The crystal structure of nanoplates and nanorods was determined by high-resolution micrographs (FEGTEM) and powder XRD methods. Figure 6a shows an FEGTEM micrograph of a group of interlocked nanoplates, captured after 120 min. The selected area electron diffraction pattern (SAED), obtained with a 30 nm electron beam diameter (Figure 6b), confirms the hcp phase of cobalt. The SAED pattern acquired by an electron beam of 5 nm diameter provides a clear spot pattern highlighting the crystalline nature and various planes of a nanoplate (Figure 6d).

Powder XRD measurements were performed to identify the crystal structure of the nanoplates. The X-ray diffraction spectrum is indexed to hcp cobalt (Figure 6e, space group 194 (*P*63/*mmc*, JCPDS card:89-4308). The planes identified through XRD are in agreement with the planes detected by SAED. The fact is emphasized by the dominant intensity of the (002) reflection in XRD in agreement with the large area of the lateral surface indexed to (002) plane in high-resolution micrograph (Figure 6c) and SAED pattern (Figure 6d). The XRD spectrum of cobalt nanoplates was collected by drying the sample directly on a glass slide in vacuum for at least 24 h. Furthermore, the analysis of the XPS spectrum of this sample shown in Figure S7 (Supporting Information File 1) shows peak broadening and therefore indicates the presence of a small amount of oxides. The oxide formation could possibly occur because of exposure to ambient atmosphere during the sample preparation process. The samples were further subjected to a controlled heat-treatment in nitrogen environment at 50 °C and 350 °C (below the phase-transformation temperature of cobalt) [38]. Figure 6e shows an increased intensity of the reflections after heat-treatment.

The nanostructures discussed here are significantly different in terms of synthesis process and XRD characterization from porous cobalt hydroxides reported in [39]. Nevertheless, the subsequent discussions indicate the formation of nanorods at higher temperatures and the use of ammonium hydroxide is a significant novelty in the growth of these nanostructures.

**Figure 7:**
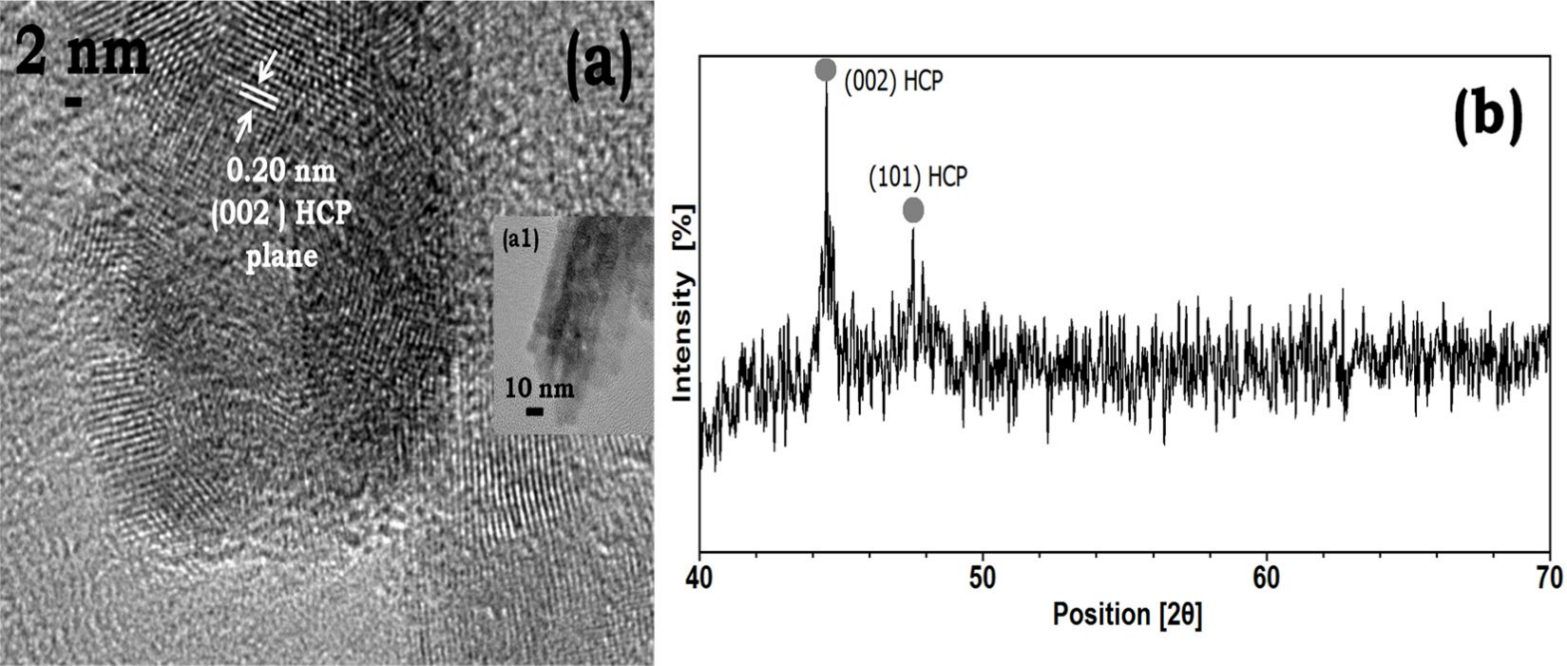
Co-TMAH nanorods obtained at 50 °C. (a) High-resolution image of a small section from inset (a1) shows a lattice spacing of 0.20 nm, the surface is perpendicular to the (002) hcp plane and the rod is polycrystalline; (b) X-ray diffraction spectrum of cobalt-TMAH-rods taken after 45 min. The larger intensity of the (002) reflection is in agreement with the lattice fringe spacing obtained along the length of the rods.

A high-resolution image of a nanorod shows grain boundaries (Figure 7a) of aggregating nanoparticles and indicates the polycrystalline nature of nanorods. A lattice spacing of 0.20 nm corresponding to the hcp Co(002) plane is obtained here. Therefore, it is likely that the presence of OH^−^ ions promotes growth normal to the (002) plane. The XRD pattern shows the crystalline nature of nanorods with two prominent reflections of the (002) and (101) planes of pure hcp cobalt (Figure 7b). The lattice spacing of the nanorods is the same as that of the flat surfaces of nanoplates obtained at room temperature (Figure 6c). But the crystalline nature is strikingly different. The surface of nanorods exhibits polycrystallinity. This can be understood from the fact that the primary particles are large (ca. 100 nm) and polycrystalline themselves, while the primary particles in the formation of nanoplates are single-crystalline. The thickness of the nanoplates is limited to two particles (size of monomers ca. 15 nm).

**Figure 8:**
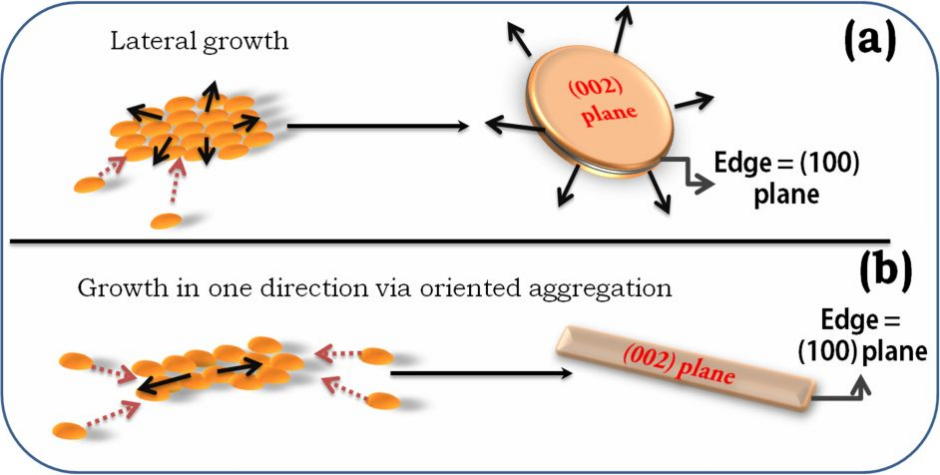
Growth under low and high driving forces. (a) Lateral growth observed under low driving force resulting in nanoplates; (b) elongated shapes are preferred at high-driving forces because of oriented aggregation of monomers. Black arrows indicate the growth direction, dotted red arrow show deposition of monomer by diffusion.

The above discussion describes the similarity in the growth direction of nanoplates and nanorods. In both nanostructures, the (002) plane remains blocked, and growth is favored perpendicular to it. Figure 8 describes the direction of monomer addition for nanoplates and nanorods while the (002) planes remain unaltered because of the adsorption of TMAH. Two-dimensional nanoplates form when the addition of monomers occurs with equal probability from all directions. During the growth of nanorods the addition of material is preferred only at extreme ends while other directions remain forbidden [40]. This change in direction of monomer addition is due to the higher temperature [40], which generates a directional anisotropy forming elongated nanostructures.

### Control of morphology

The shape of the cobalt nanostructures was modified by replacing TMAH with NH_4_OH, TEAH and TBAH. These compounds have an increasing cationic diameter and can demonstrate the effect of the cationic diameter on the formation of nanostructures.

**Figure 9:**
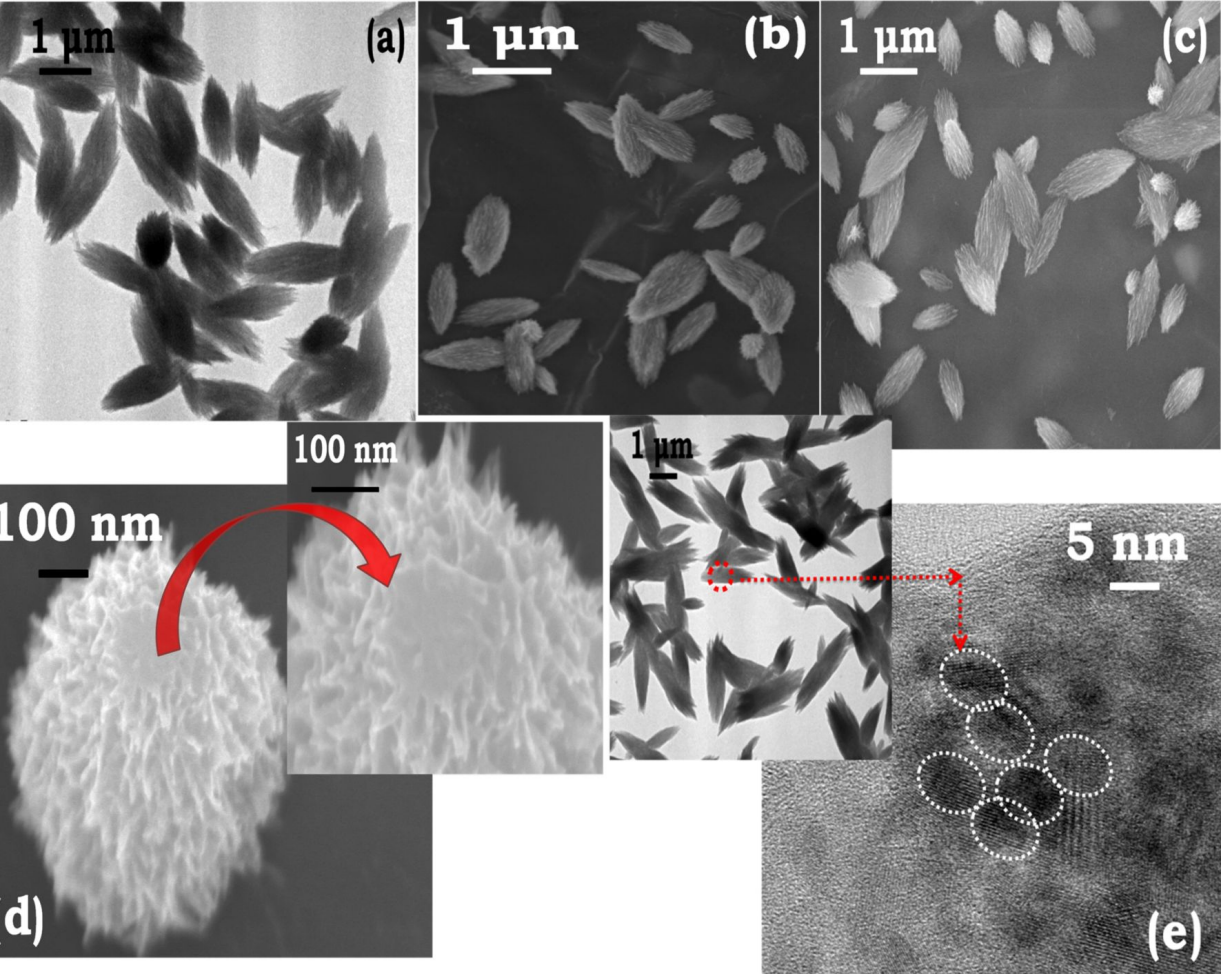
Cobalt structures formed in the presence of NH_4_OH. (a,b) FEGTEM and FEGSEM micrographs captured after 15 min of ageing show spindles with length *l* = 900 nm and diameter *d* = 200 nm; (c) FEGSEM micrograph captured after 45 min of ageing shows spindles with length *l* = 1100 nm and diameter *d* = 230 nm; (d) FEGSEM micrograph of a single spindle standing on its tip; the figure shows irregular corrugations on the surface of the spindle; (e) high-resolution FEGTEM micrograph of a spindle.

The ultrasonication of cobalt seeds with NH_4_OH yielded unique spindle-shaped nanostructures after 15 min (Figure 9a,b) with a typical length of 900 nm and a diameter of 200 nm. The spindles grow to an average length of 1100 nm and an average diameter of 230 nm after 45 minutes (Figure 9c). It is interesting to note that the formation of spindles in the presence of NH_4_OH is faster (15 min) compared to the formation of nanoplates using TMAH (45 min).

NH_4_OH is often used as a crystallization modifier and adsorbs preferentially on certain crystal planes. Cone-shaped nanostructures of cobalt were obtained by using NH_4_OH during electrodeposition [26]. The growth of these unique cone nanostructures is initiated by screw dislocations. In this study, interesting spindles with peculiar conical edges are synthesized that can be, in principle, surface-functionalized for various applications. A magnified image of the tip of a single spindle in its upright position shows irregular features on the surface and a blunt tip (Figure 9d). A high-resolution image (Figure 9e) shows that the spindles are formed by aggregation of spherical nanoparticles, comparable to the formation of nanoplates or nanorods. There are significant differences though, namely, the time-scale of spindle formation and the diameter of the spindle. The available primary cobalt particles progressively decrease and their influx towards the growth site, should be limited because of steric hindrance by the adsorption agent. This is proposed based on the fact that the radius of gyration of NH_4_^+^ is 0.90 Å [41], whereas the radius of gyration and the ionic radius of TMA^+^ are larger, namely, 1.93 Å and 2.9 Å, respectively [41–42].

**Figure 10:**
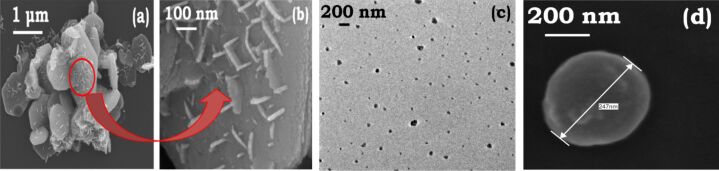
Manipulation of morphologies by TEAH and TBAH. (a,b) FEGSEM micrographs show cobalt-TEAH captured after 45 min. Micrographs show that small nanoplates of 70 nm diameter are formed on a bigger hexagonal base plate with typical 1000 nm edge length; (c,d) FEGTEM and FEGSEM micrographs captured after 45 min of the cobalt-TBAH system show the formation of nearly spherical nanoparticles. The presence of larger cation inhibits anisotropic growth in the TBAH system.

The shape of the anisotropic cobalt nanostructures was next controlled by using additives with longer alkyl chains, TEAH and TBAH. As the length of the alkyl chain increases, the dissociation of OH^−^ ions decreases and affects the morphology thus achieved. The use of TEAH and TBAH generates particles with distinct surface topology and crystalline properties. Using TEAH, small nanoplates of approximately 50–70 nm with a thickness of 15 nm (Figure 10a) are obtained. The interlocking is much lower than that observed when TMAH is used. The corresponding FEGSEM images (Figure 10b) also show that these nanoplates form decorated structures on large hierarchical hexagonal base-plates with edge lengths of around 1000 nm. The addition of TBAH yields nearly spherical nanoparticles with an average size of 250 nm (Figure 10c,d) suggesting isotropic growth of the crystals. The dissociation of hydroxide ions from TBAH is the least and TBA^+^ has the largest ionic radius of the additives used here. A general stability, in terms of shape, size and color has been observed over 15–20 days for the all samples with controlled exposure to ambient conditions.

### Magnetic characterization

**Figure 11:**
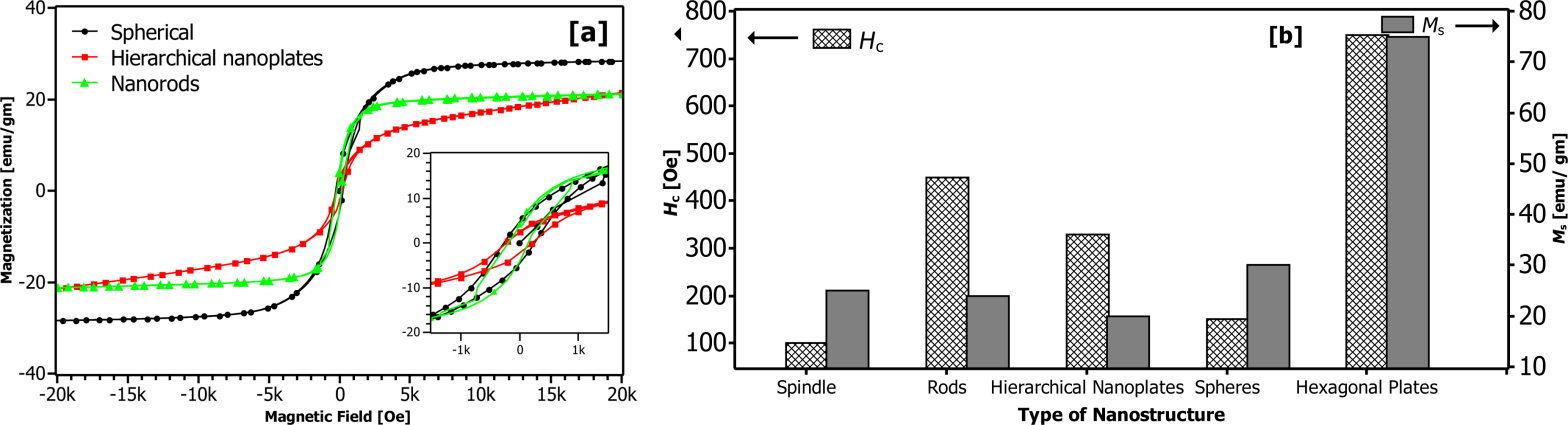
Magnetic properties of the synthesized nanostructures. (a) Magnetization versus magnetic field recorded at 300 K for Co-TMAH nanoplates, Co-TMAH rods synthesized at 50 °C, Co-TEAH hierarchical plates; (b) summary of saturation magnetization (*M*_s_) and coercivity (*H*_c_) of the nanostructures.

To investigate the effect of microstructure and shape on the magnetic properties, magnetization measurements of various cobalt hierarchical structures were carried out at room temperature in an applied magnetic field of 10 kOe. The magnetization plots for interlocked nanoplates, rods and hierarchical plates are shown in Figure 11a; the inset presents enlarged *M*–*H* loops. All nanostructures show ferromagnetic behavior. The magnetic properties of all synthesized nanostructures are summarized in Figure 11b.

The values of saturation magnetization (*M*_s_) and coercivity (*H*_c_) depend on the shape of the nanostructures. The nanoplates show *M*_s_ of 20 emu/g and *H*_c_ of ca. 330 Oe (Figure 11b). Compared to the coercivity value of bulk cobalt (a few tens of oersteds at room temperature [43]), the enhanced coercivity value here is attributed to the two-dimensional anisotropic shape [44–45]. The saturation magnetization, however, is small compared to the bulk value of 168 emu/g [43]. The small value of *M*_s_ is likely due to formation of oxides as indicated in the XPS spectrum shown in Figure S7 (Supporting Information File 1). Oxide formation was inevitable since the analyses required drying or separation of cobalt nanoparticles that might lead to interactions of the surfactant with metal surfaces [46].

Polycrystalline nanorods show a higher value of *M*_s_ than nanoplates (20 emu/g) and a large coercivity (*H*_c_) of 450 Oe (Figure 11b). Hierarchical hexagonal cobalt plates synthesized in TEAH exhibit high values of *M*_s_ and *H*_c_ of 75 emu/g and 750 Oe, respectively.

It is worth mentioning that the coercivity of these nanostructures is significantly higher than that obtained in various similar hierarchical nanostructures [27]. Asymmetry in the shape and the larger dimensions contribute towards significant enhancement in the magnetic characteristics. Higher coercivity is a prerequisite for high-density information storage and permanent magnets. More investigations are still in progress to improve the coercivity and other magnetic properties of the nanostructures.

## Conclusion

In the present work, we show that the quaternary ammonium compounds produce hierarchical complex nanostructures of cobalt. Electron microscopy effectively reveals that the anisotropic shapes are an outcome of agglomeration of initial spherical seed nanoparticles the aggregation of which is facilitated by particle–particle collisions, magnetic moment and directed by the presence of a stabilizing agent (such as TMAH, NH_4_OH, TEAH, or TBAH), yielding different nanostructures. The magnetic properties of the nanostructures depend on microstructure and shape.

The method significantly advances the existing repository of nanoparticle synthesis by its easy handling parameters, synthesis in aqueous medium, and effective control of morphology. The study opens several roads for understanding the growth of highly anisotropic nanostructures without the aid of complex microscopy accessories. The importance of the combined interpretation of FEGTEM/HRTEM and FEGSEM micrographs is also emphasized. The method proposed is simple and the results should be useful for designing a controlled synthesis of complex magnetic nanostructures for various technological applications.

## Supporting Information

File 1 Additional experimental data.
