# Supplementary material for: Formation and shape-control of hierarchical cobalt nanostructures using quaternary ammonium salts in aqueous media
Source: Beilstein J Nanotechnol. 2017 Feb 23;8:494–505. doi: 10.3762/bjnano.8.53 (PMC5331312; doi:10.3762/bjnano.8.53)
Supplement: File 1 — Additional experimental data. [file Beilstein_J_Nanotechnol-08-494-s001.pdf]

Supporting information for:

Formation and shape-control of hierarchical cobalt nanostructures using quaternary ammonium salts in aqueous media

Ruchi Deshmukh, Anurag Mehra and Rochish Thaokar*

Department of Chemical Engineering, Indian Institute of Technology Bombay, Mumbai, India

E-mail: rochish@iitb.ac.in

Phone: +91-22-2572-2545. Fax: +91-22-2572-3480

Experimental Section: Characterization of Cobalt seeds

A three step-protocol was followed to synthesize cobalt anisotropic nanostructures. Typical synthesis involves direct reduction of a cobalt salt precursor- cobalt chloride hexahydrate ($CoCl_2.6H_2O$ (0.8 mL of 0.4 M solution) by sodium borohydride ($NaBH_4$) (100 mL of 0.1 M), both prepared in deaerated MilliQ water. The reaction was carried out under ultrasonication bath and the resulting black precipitate was collected using a strong permanent magnet. The ultrasonication bath temperature was maintained at $28\pm2°C$ and total reduction time in all cases was 3 minutes. The precipitate thus obtained was further washed with ethanol-water mixture, at least four times, to remove traces of secondary species of reaction such as, sodium, boron etc. An energy dispersive spectrum (EDS) shows reduction of the initially observed peaks for boron (B) and sodium (Na) after treating the sample with ethanol-water

(Figure S1a, and S1b). The precipitate here after is called ”seed” of cobalt with size around 30-50 nm.

The seeds after ethanol-water mixture were further treated with either- TMAH (2.7 M), TEAH (2.3 M), TBAH (1.5 M) or NH_4OH (1.8 M) as shape selection agents. An amount of 15 mL of these additives was added to the entire precipitate collected after the ethanol-water washing. The seeds were treated with ionic liquid in a closed round-bottom flask in an ultrasonic bath. The total run time for ultrasonication was 20 minutes, and bath temperature was held constant at 28±2°C.

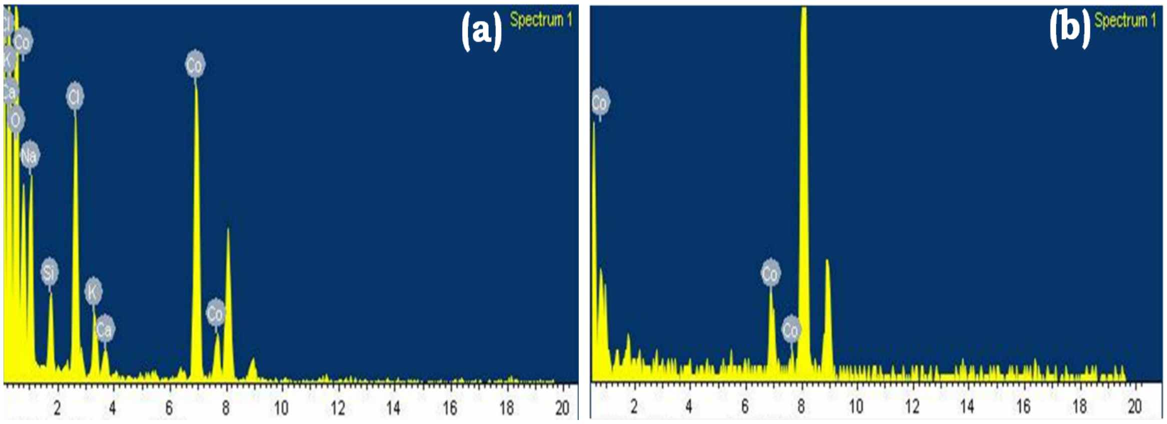

Figure S1: Energy dispersive spectrum (EDS) of cobalt seeds at different stages: (a) EDS of synthesized cobalt-seeds, before washing with (ethanol-water) mixture, (b) EDS of cobalt seeds after magnetic decantation and (ethanol - water) washing.

Although the chemistry of cobalt reduction by borohydride is complicated, it is well established that the aerobic handling promotes a sacrificial reaction in which the secondary product of Co_2B oxidizes while Co(s) is reduced (Glavee et al., 1992; Salgueirio-Maceira et al., 2006), yielding metallic cobalt. The state of particles obtained by this method was observed to be dependent on factors such as, age of the solutions, time taken for mixing the reagents etc. Only freshly prepared solutions led to reproducible results, moreover a quick mixing of the reagents was found to be essential to achieve the above mentioned size range

of 30-50 nm. The reduction reaction (Glavee et al., 1992) follows:

$$2\,\mathrm{CoCl_2} + 4\,\mathrm{NaBH_4} + 9\,\mathrm{H_2O} \rightarrow \mathrm{Co_2B} + 4\,\mathrm{NaCl} + 12.5\,\mathrm{H_2} + 3\,\mathrm{B(OH)_2} \tag{1}$$

$$4\,\mathrm{Co_2B} + 3\,\mathrm{O_2} \longrightarrow 8\,\mathrm{Co} + 2\,\mathrm{B_2O_3} \tag{2}$$

The high-resolution micrograph of cobalt seeds after ethanol-water treatment and magnetic decantation shows well formed spherical nanoparticles (Figure S2a). A lattice spacing of 0.10 nm is obtained that corresponds to the (110) HCP plane of cobalt (Figure S2b). SAED pattern in inset S2b here shows crystallinity in sample.

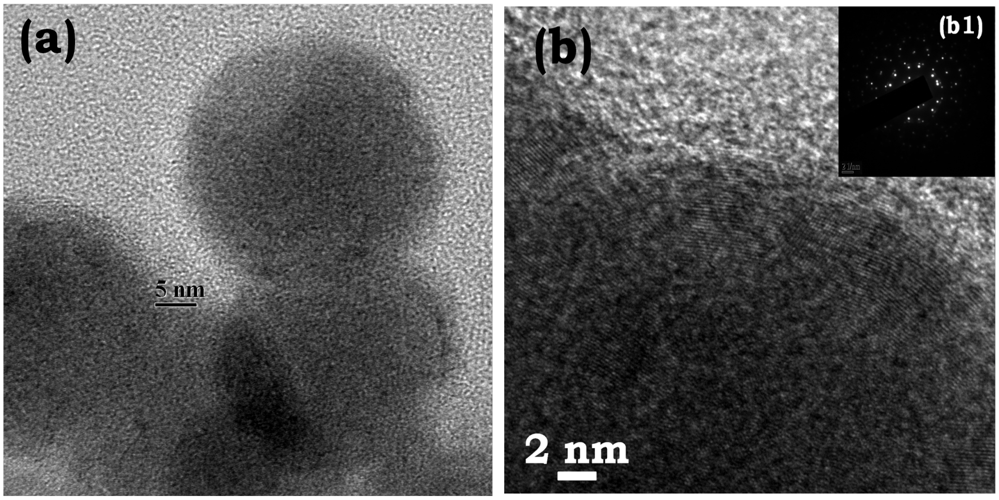

Figure S2: (a) Cobalt seeds obtained after magnetic decantation, (b) high-resolution image of seed particle, average d-spacing calculated is 0.12 nm- (110) HCP plane.

Size distribution of Co-seeds

Typical seed size was calculated as 29±3 nm. The size distribution of seeds is shown in following Figure S3b is monomodal

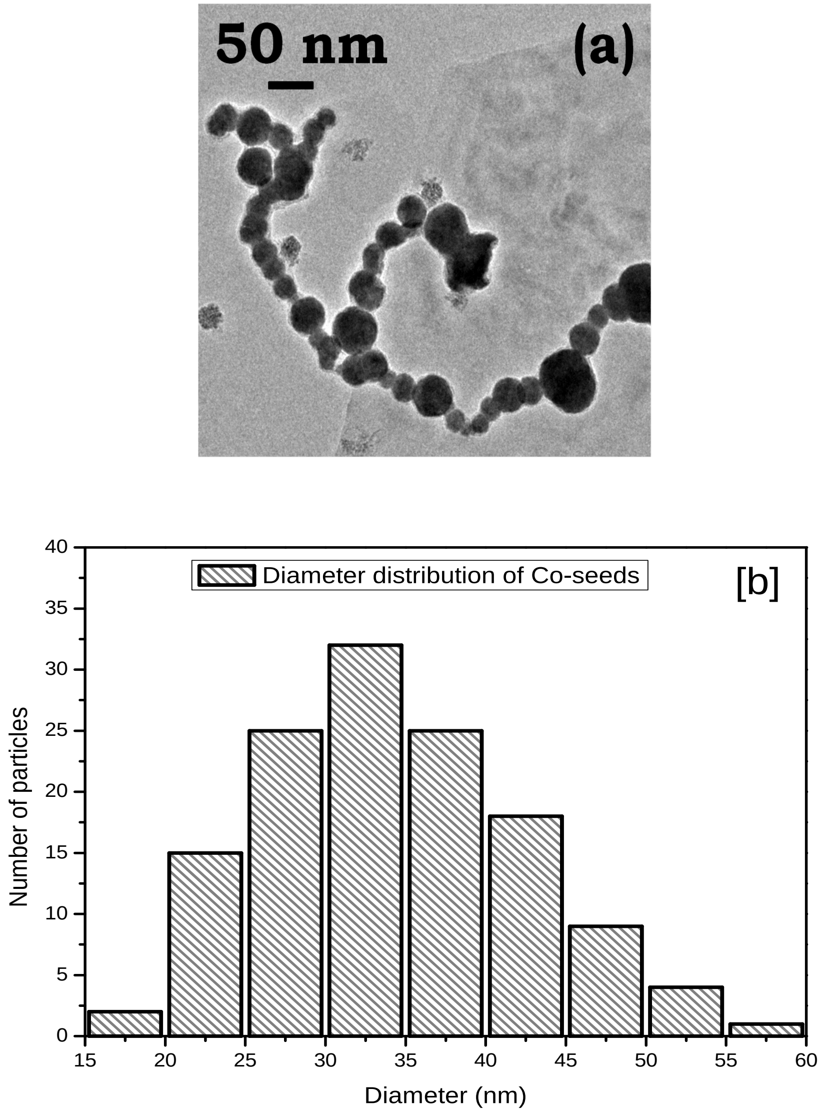

Figure S3: (a) HRTEM Micrograph and (b) size distribution of Co-seeds obtained after magnetic decantation and ethanol+water washing, average seed size is 29±3 nm.

These seeds were further ultrasonicated for 10 minutes, with different stabilizing agents, such as tetra*alkyl*ammonium hydroxide, (TRAOH, where R is: methyl, ethyl or butyl) or with ammonium hydroxide (NH_4OH). The transients of the process is studied by capturing

electron micrographs at different time in the growth regime, and is reported in the main paper.

Energy dispersive spectrum (EDS) of Cobalt nanoplates

Energy dispersive spectrum (EDS) taken at 120 minutes on a group of nanoplates show the cobalt peak (Figure S4). Other dominant peaks are for microscopy grid made of copper mesh.

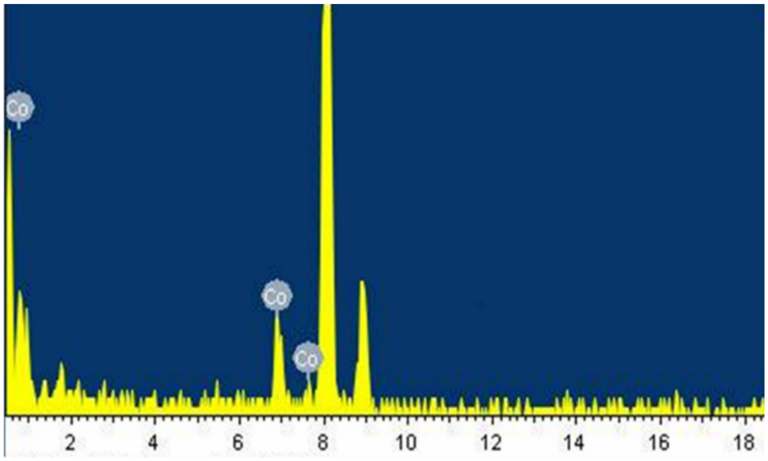

Figure S4: Energy dispersive spectrum (EDS) of Co-TMAH nanoplates in growth regime: spectrum captured by FEGTEM at 45 minutes.

Addition of tetra-*methyl*-ammonium chloride (TMACL)

In a set of experiment, tetra*methyl* ammonium chloride (TMACL 2.7 M, pH=8.5) was added in place of TMAH. Addition of TMACL yields only randomly aggregated structures. Well formed anisotropic nanostructures were not observed even after leaving cobalt-TMACL sample for 2 weeks (Figure S5). Hence, it is proposed that the alkyl group TMA^+ independently could not generate anisotropy, and was effective only in presence of optimum concentration of hydroxide OH^-.

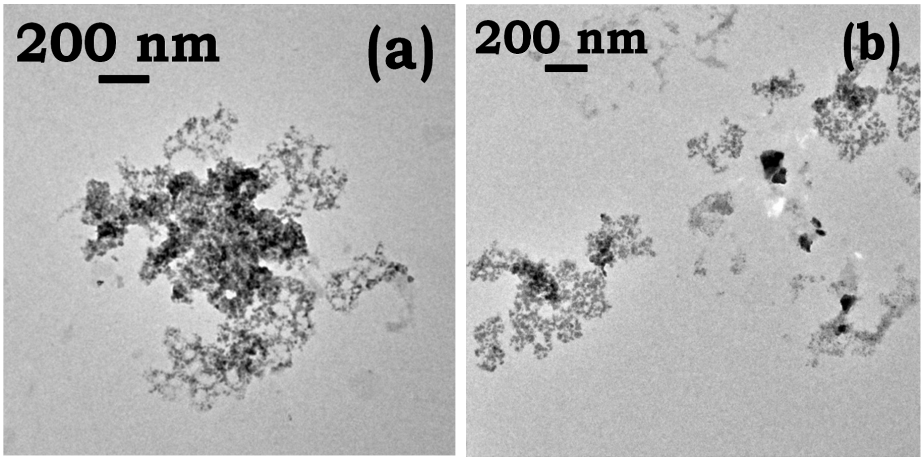

Figure S5: Cobalt-TMACL (pH 8.5), anisotropic nanoparticles were not observed even after aging the sample for 2 weeks: (a) micrograph of freshly prepared sample, (b) micrograph captured after ageing the sample for 2 weeks.

Co-TMAH: Micrographs of twinned particles at zero minute

Additional micrographs indicating the twinning at zero minute is shown in following Figure S6. Majority of particles at zero-time possessed twinned structure.

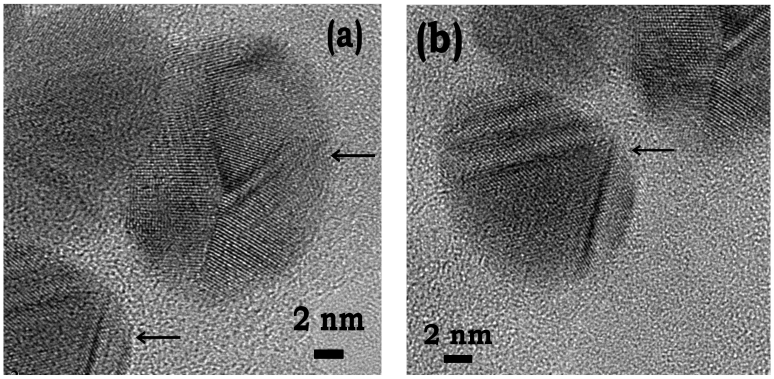

Figure S6: Various micrographs showing the twinning observed in nanoparticles at zero minutes, black arrows indicate the twinning defect.

Surface characterization by X-ray photoelectron spectroscopy

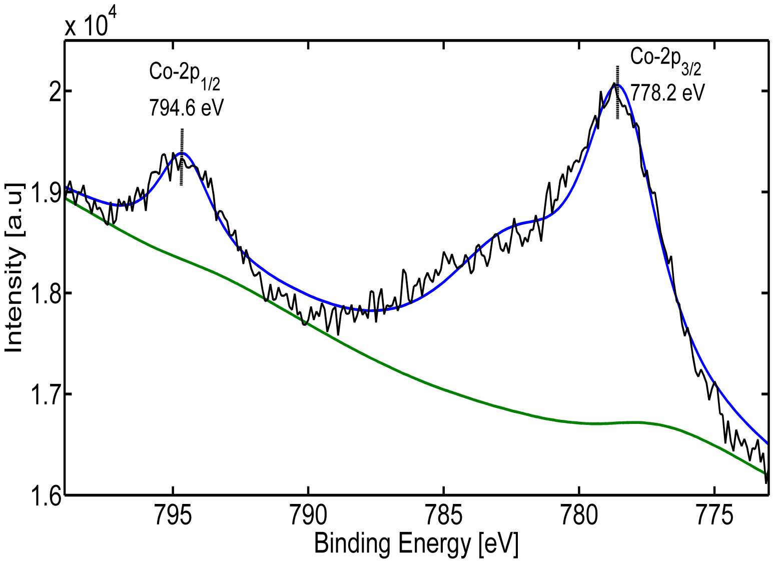

Figure S7: X-ray photoelectron spectrum cobalt (2p). Positions 778.2 eV and 794.6 eV correspond to Co-2$p_{3/2}$ and Co-2$p_{1/2}$ peaks. The peak broadening indicates presence of oxide in the sample (Farr and Griesser, 1989; Li et al., 2004; Li and Liao, 2008).

References

Glavee, G.; Klabunde, K.; Sorensen, C.; Hadjapanayis, G. *Langmuir* **1992**, *8*, 771–773.

Salgueirio-Maceira, V.; Correa-Duarte, M.; Farle, M.; Lopez-Quintela, M.; Sieradzki, K.; Diaz, R. *Langmuir* **2006**, *22*, 1455–1458.

Farr, N. G.; Griesser, H. J. *Journal of Electron Spectroscopy and Related Phenomena* **1989**, *49*, 293 – 302.

Li, J.; Qin, Y.; Kou, X.; He, H.; Song, D. *Materials Letters* **2004**, *58*, 2506 – 2509.

Li, H.; Liao, S. *Journal of Physics D: Applied Physics* **2008**, *41*.
